# Formaldehyde exposure induces differentiation of regulatory T cells via the NFAT-mediated T cell receptor signalling pathway in Yucatan minipigs

**DOI:** 10.1038/s41598-022-12183-8

**Published:** 2022-05-17

**Authors:** Jeongsik Park, Goo-Hwa Kang, Youngkyu Kim, Ju Young Lee, Jeong Ah Song, Jeong Ho Hwang

**Affiliations:** 1 Animal Model Research Group, Jeonbuk Department of Inhalation Research, Korea Institute of Toxicology, 30 Baehak 1-gil, Jeonguep, Jeollabuk-do 56212 Republic of Korea; 2 Department of Stem Cell and Regenerative Biotechnology, Konkuk University, 120 Neungdong-ro, Gwangjin-gu, Seoul-si, 27447 Republic of Korea; 3 Department of Human and Environmental Toxicology, University of Science & Technology, Daejeon, 34113 Republic of Korea

**Keywords:** Immunology, Adaptive immunity, Cellular immunity, Health care, Occupational health

## Abstract

The use of minipigs (*Sus scrofa*) as a platform for toxicological and pharmacological research is well established. In the present study, we investigated the effect of formaldehyde (FA) exposure on helper T cell-mediated splenic immune responses in Yucatan minipigs. The minipigs were exposed to different inhaled concentrations of FA (0, 2.16, 4.62, or 10.48 mg/m^3^) for a period of 2 weeks. Immune responses elicited by exposure to FA were determined by assessing physiological parameters, mRNA expression, and cytokine production. Additionally, the distribution of helper T cells and regulatory T (Treg) cells and expression of NFAT families, which are well-known T cell receptor signalling proteins associated with regulatory T cell development, were evaluated. Exposure to FA suppressed the expression of genes associated with Th1 and Th2 cells in minipigs in a concentration-dependent manner. The subsequent production of cytokines also declined post-FA exposure. Furthermore, exposure to FA induced the differentiation of CD4^+^ Foxp3^+^ Treg cells with divergent expression levels of NFAT1 and NFAT2. These results indicated that exposure to FA increased the Treg cell population via the NFAT-mediated T cell receptor signalling pathway, leading to suppression of effector T cell activity with a decline in T cell-related cytokine production.

## Introduction

Minipigs (*Sus scrofa*) have been extensively reported for their value and utility as an animal model for research^1^. In the European Union, more than 60,000 minipigs are used every year for scientific research, including surgical, physiological, and biomedical research^2^. There has been a notable interest in minipigs owing to the anatomical, physiological, and biochemical similarities between minipigs and humans^1–4^, particularly in terms of the nasal cavity, skin, and heart^2,3^. Several sequence studies have reported that the evolutionary distance between pigs and humans is smaller than that between rodents and humans^5–7^, indicating that the pig genome is fairly homologous with the human genome. Recently, several human diseases that could not be reflected in a rodent model have been successfully modelled in pigs through genetic modification^8,9^. Additionally, the use of minipigs in toxicological and pharmacological research, including in the analysis of pulmonary exposure to chemicals and drugs, has recently been reported^10–13^.

Formaldehyde (FA), a colourless, flammable, strongly reactive chemical present in homes and other buildings, is a common indoor and outdoor pollutant^14^. FA is a common component of pressed-wood products, urea-FA insulations, and glues and adhesives^15^, and is also widely present in plastics, cosmetics, industrial fungicides, and disinfectants^16^. Thus, individuals can be subjected to high levels of FA via inhalation at their homes or workplaces. Additionally, FA is recognised as a toxic chemical at certain concentrations (80–2000 ppb) and the health risks are increased at room temperature owing to the volatile nature of FA^17,18^. The International Agency for Research on Cancer has classified FA as a category-1 human and animal carcinogen responsible for nasopharyngeal cancer and leukaemia^19,20^. According to a toxicological review by the US Environmental Protection Agency, FA induces adverse events, including sensory irritation and immuno-, neuro-, and developmental toxicity^21^. Recent human and animal studies have indicated that immune dysregulation caused by FA exposure may aggravate allergic inflammation^22,23^. Conversely, immunosuppression caused by FA exposure may lead to cancer progression^24–26^. These studies indicate that FA exerts potential toxic effects on the immune system.

The association between FA exposure and asthma has been investigated by several studies^17,27–31^. Jung et al. showed that exposure to FA induces and aggravates airway inflammation by promoting eosinophil infiltration and T cell-related cytokine production^32^. Conversely, recent studies have indicated that FA-exposed rodents sensitised with ovalbumin exhibited impaired development of allergic responses, along with reduced T cell-related cytokine production, bronchial responsiveness, and mast cell activation^17,27^. FA also affects the different types of T cells, including CD4^+^ T cells, CD8^+^ T cells, and memory T cells^33–35^. Sandikci et al. reported that the levels of CD4^+^ and CD8^+^ T cells were increased in adult rats exposed to FA^34^. Aydin et al. demonstrated that the absolute number and percentage of T cells significantly increased in the blood of fibreboard-producing plant workers who had been exposed to FA^35^. In contrast, Hosgood et al. reported that the counts of CD4^+^ and CD8^+^ T cells were significantly low in workers exposed to FA^33^. While the reason for the conflicting data between human and animal studies remains unclear, previous reports have, overall, stressed the need for further research assessing the potential toxic effects of FA exposure on the immune system.

CD4^+^ helper T cells play crucial roles in host health and immune-mediated disease^36^. They can differentiate into various subsets of effector T cells (Th1 and Th2) and regulatory T (Treg) cells after activation^37^. The three subsets express distinct cytokine signatures, master transcription factors, and homing receptors in response to infection by pathogens^36^. Th1 cells are characterised by the expression of interferon-γ (IFN-γ), interleukin (IL)-2, and tumour necrosis factor-α (TNF-α)^37^. These cells are particularly important in the defence against intracellular bacteria, such as *Listeria* and *Mycobacterium tuberculosis*, but can also exacerbate the development of organ-specific autoimmune diseases and chronic inflammatory disorders^38^. Th2 cells express IL-4, IL-5, and IL-13 and are immunologically active against extracellular pathogens, such as worms^39^. Th2 cells also promote acute and chronic inflammatory responses against a myriad of allergens^40^. Treg cells are characterised by the expression of the forkhead transcription factor, *Foxp3*, which is essential for their development and suppressive functions^41^. These cells play a critical role in maintaining the homeostasis of the immune system, regulating effector T cell responses and preventing autoimmune reactivity^42^. However, an excessive presence of Treg cells can have a detrimental effect on the host due to potentially increasing susceptibility to opportunistic infections and inhibition of antitumor immunity^43–45^.

Several studies have investigated the relationship between FA exposure and immune responses in rodent model systems. However, FA exposure studies using minipigs as a non-rodent animal model have not yet been reported. In the present study, we used Yucatan minipigs to investigate the effects of FA exposure on the immune system by evaluating the helper T cell and Treg cell populations and expression of immune-related factors, including cytokines. Therefore, the aim of this study is to offer insight into the underlying mechanism orchestrating the FA exposure-induced immune modulation that has detrimental health effects, such as opportunistic infections and cancer development.

## Results

### FA exposure does not induce a change in body or organ weight

**Figure 1:**
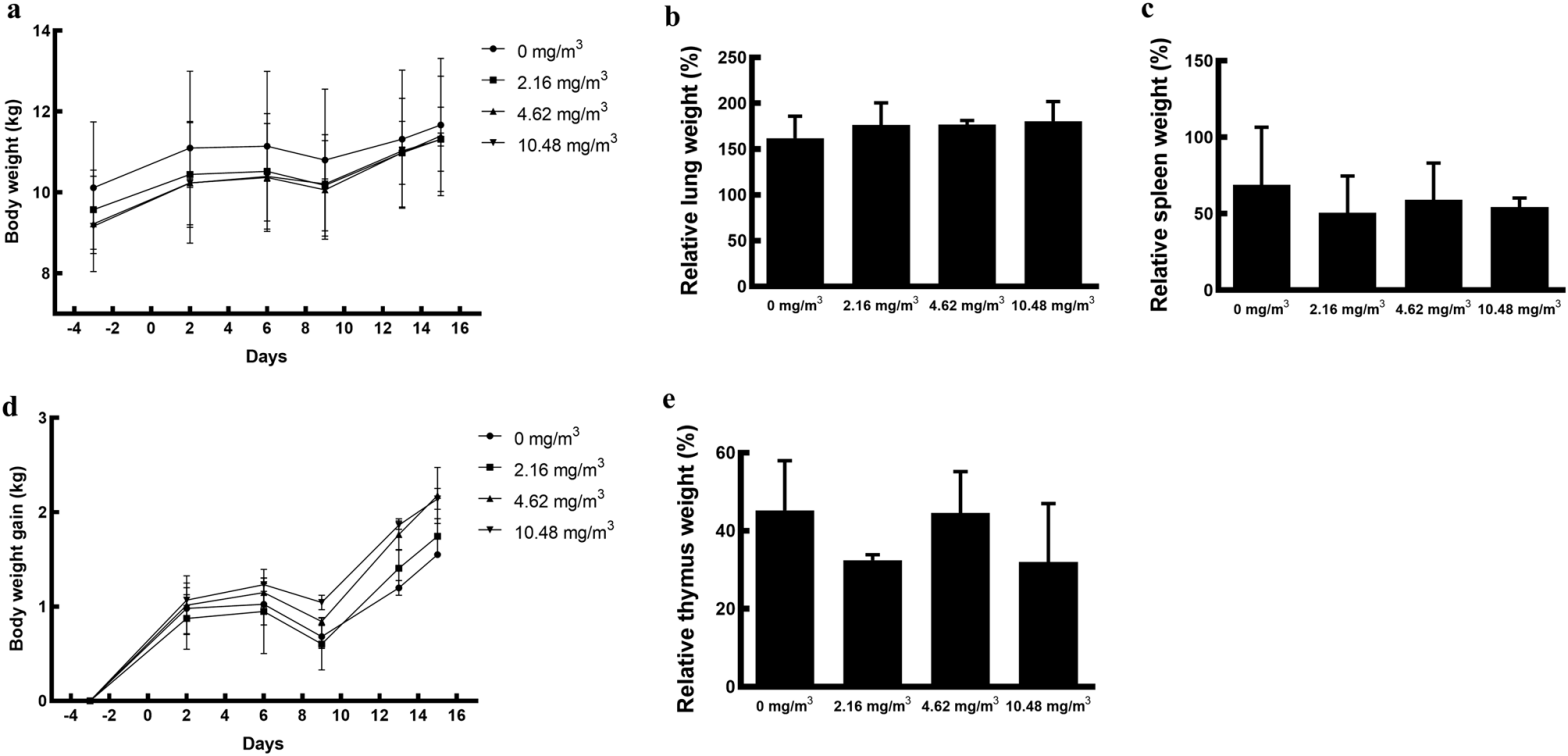
Body and organ weights in Yucatan minipigs. Changes in body weight (**a**) and weight gain (**d**) of minipigs exposed to FA. Relative weights of organs, including the lung (**b**), spleen (**c**), and thymus (**e**) were calculated. Data are presented as the mean ± SD (*n* = 2 minipigs/group). *FA* formaldehyde.

Exposure to FA did not cause any differences in the body weights (*p* > 0.918) of or any weight gain (*p* > 0.444) in the minipigs in the FA-exposed group (Fig. 1a,d). In the control group exposed to clean air, the body weights increased from 10.1 ± 1.6 kg to 11.7 ± 1.6 kg. All animals in the FA-exposed group exhibited similar changes in body weight with time as those in the control group. We also assessed the effect of FA exposure on the relative organ weight in minipigs in the following manner.

$${\text {Relative\,organ\,weight}} = {\text{organ\,weight}}/{\text{brain\,weight}} \times 100\%.$$

There were no significant changes in the relative weights of organs, including the lung, spleen, and thymus [lung (control group: 161.0 ± 24.0%, 2.16 mg/m^3^ FA exposure group: 176.4 ± 7.6%, 4.62 mg/m^3^ FA exposure group: 176.7 ± 4.6%, 10.48 mg/m^3^ FA exposure group: 180.5 ± 21.5%, *p* > 0.675); spleen (control group: 68.9 ± 37.7%, 2.16 mg/m^3^ FA exposure group: 50.5 ± 24.2%, 4.62 mg/m^3^ FA exposure group: 59.2 ± 24.0%, 10.48 mg/m^3^ FA exposure group: 54.3 ± 6.0%, *p* > 0.884); and thymus (control group: 45.2 ± 12.7%, 2.16 mg/m^3^ FA exposure group: 32.5 ± 1.4%, 4.62 mg/m^3^ FA exposure group: 44.5 ± 10.6%, 10.48 mg/m^3^ FA exposure group: 32.0 ± 14.9%, *p* > 0.668)] (Fig. 1b,c and e).

### FA exposure causes a decline in IFN-γ, TNF-α, and IL-4 production

**Figure 2:**
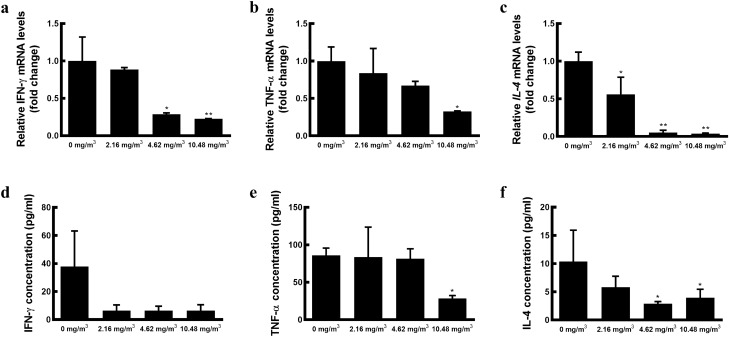
Helper T cell-related mRNA expression and cytokine production by FA exposure. Splenocytes isolated from FA-exposed minipigs were cultured with 2.5 μg/mL Concanavalin A (Con A) for 72 h. The mRNA expression of genes associated with Th1 (*IFN-γ, TNF-α*) and Th2 (*IL-4*) was evaluated by qRT-PCR (**a**–**c**). Target gene expression was normalised to *GAPDH* expression and the expression is presented as fold change relative to the control group. Production of cytokines associated with Th1 (IFN-γ, TNF-α) and Th2 (IL-4) was evaluated (**d**–**f**). Data are presented as the mean ± SD from two independent experiments (*n* = 2 minipigs/group). *p < 0.05, **p < 0.01. *FA* formaldehyde.

To investigate the effect of FA exposure on the immune system, we assessed the expression of genes associated with Th1 (*IFN-γ, TNF-α*) and Th2 (*IL-4*) cells and the subsequent production of their cytokines. Exposure to FA resulted in a decline in *IFN-γ, TNF-α,* and *IL-4* mRNA levels in a concentration-dependent manner, with significant suppression of *IL-4* expression observed at all assessed FA levels. Additionally, gene expression was significantly lower in the animals exposed to 10.48 ± 0.64 mg/m^3^ FA than in the control group animals (Fig. 2a–c). A similar expression pattern was observed in the production of Th1- and Th2-related cytokines; however, the production of IL-4 (*p* = 0.02) and TNF-α (*p* = 0.02) was significantly lower in the FA exposure groups than in the control group (Fig. 2d–f).

### FA exposure promotes an increase in Treg cell population

**Figure 3:**
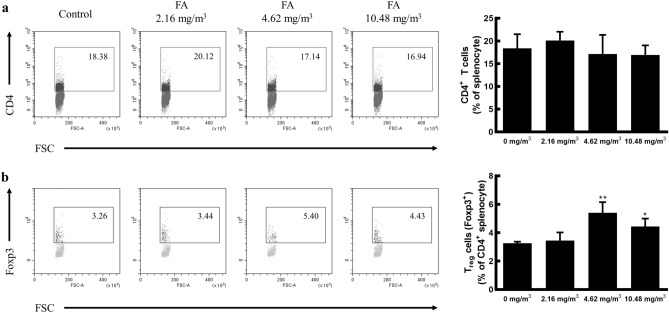
Regulation of the CD4^+^ T cell and Treg cell populations by FA exposure. Single spleen cells isolated from minipigs exposed to FA were stained with PerCP-Cy™5.5-conjugated anti-CD4 and FITC-conjugated anti-Foxp3 monoclonal antibodies. The fluorescence levels were measured using a CytoFlex flow cytometer. (**a**) CD4^+^ T cell population in single spleen cells. (**b**) CD4^+^ Foxp3^+^ Treg cell population in CD4^+^ T cells. Data are shown as representative dot plots and presented as the mean ± SD from two independent experiments (*n* = 2 minipigs/group). *p < 0.05, **p < 0.01. *FA* formaldehyde.

Exposure of minipigs to FA caused no significant differences in the CD4^+^ helper T cell population size relative to that of the control group (control group: 18.38%, 2.16 mg/m^3^ FA exposure group: 20.12%, 4.62 mg/m^3^ FA exposure group: 17.14%, 10.48 mg/m^3^ FA exposure group: 16.94%, *p* = 0.443). However, the Treg cell population (CD4^+^ Foxp3^+^ cells) was found to be significantly increased from 3.26% (control) to 5.40% and 4.43% following exposure to 4.62 mg/m^3^ and 10.48 mg/m^3^ of FA, respectively (Fig. 3).

### FA exposure results in increased NFAT1 expression and decreased NFAT2 expression

**Figure 4:**
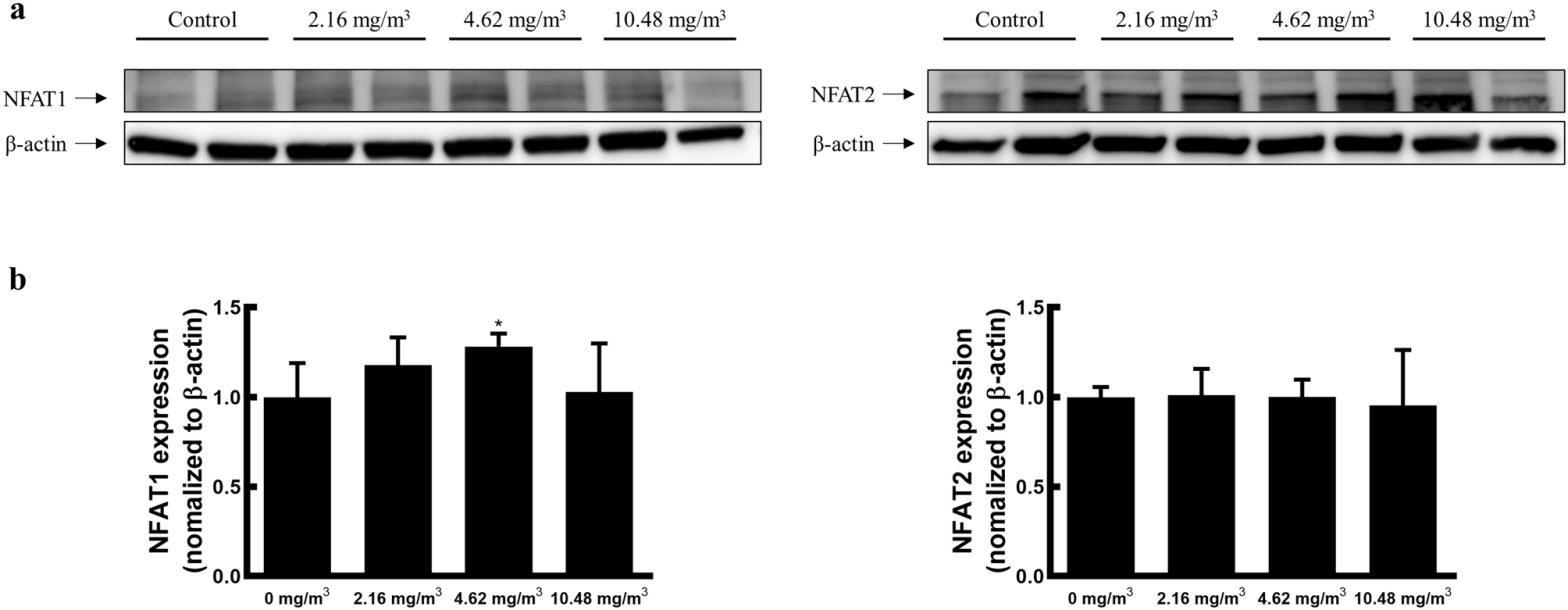
Expression of NFAT1 and NFAT2 in the spleen after FA exposure. (**a**) NFAT1 and NFAT2 expression assessed via western blotting. Gel was cut prior to transfer onto PVDF membranes, and each band from the same gel was grouped together. The original raw data are shown in Supplementary information. (**b**) Densitometric analysis of the blots. The target protein expression levels were normalised to β-actin expression. Data are presented as the mean ± SD from two independent experiments (*n* = 2 minipigs/group). *p < 0.05. *FA* formaldehyde.

Nuclear factor of activated T cells (NFAT) families are well-known T cell receptor (TCR) signalling proteins important for the regulation of Treg cell development^42^. To investigate the molecular mechanism of action of Treg cells in T cell-related cytokine suppression due to FA exposure, we evaluated the protein expression of NFAT1 and NFAT2. Notably, NFAT1 expression in the 4.62 mg/m^3^ FA exposure group was nearly 1.28-fold higher than that in the control group. In contrast, NFAT2 expression in the FA exposure groups was decreased by approximately 0.94-fold compared with that in the control group (Fig. 4).

## Discussion

Minipigs, which bear close similarity to humans, are a useful non-rodent animal model for toxicology research studies. This is the first study on minipigs addressing the issue of FA exposure-induced immune modulation. Many studies have reported that FA exposure affects helper T cell-related immune responses. However, the role of Treg cells in FA exposure-induced immune responses is still not well understood.

FA is a ubiquitous environmental pollutant and its ingestion by inhalation constitutes occupational and environmental health hazards^47^. In the present study, we report the suppressive effects of FA exposure on splenic immune responses, determined by evaluating the helper T cell and Treg cell populations and by assessing the expression of immune-related factors including mRNAs, cytokines, and proteins in Yucatan minipigs. We administered FA at a concentration of 2.16 mg/m^3^ in this study based on the no-observed-adverse-effect-concentration (for mouse: 2.46 mg/m^3^, for monkey: 1.23 mg/m^3^) assigned by the Organisation for Economic Co-operation and Development Screening Information Dataset (OECD SIDS)^48^. The current short-term exposure limit for FA in the United States is 2.46 mg/m^3^^49^. We also assessed the effect of FA at a concentration of 4.62 mg/m^3^, as it was the lowest-observed-adverse-effect l concentration (for mouse: 5.04 mg/m^3^, for monkey: 3.69–7.38 mg/m^3^) as determined by the OECD SIDS^48^. Finally, a concentration of 10.48 mg/m^3^ FA was used as the optimal high concentration to continuously control the minipig inhalation system. A previous animal study using these concentrations reported that FA exposure impaired the function and differentiation of natural killer cells^50^. Other studies have also shown that FA exposure affects immune responses, including helper T cells and lung inflammation-related up- and down-regulation of gene and protein expression^32,51,52^. Additionally, the mouse FA concentrations used in several studies were converted into minipig FA concentrations using Alexander's formula^53^; these results and the common ratio of 2 are reflected in our exposure concentrations (Park et al.^46^: 1.38, 5.36 mg/m^3^ → 0.84, 3.27 mg/m^3^; Tarkowski et al.^54^: 2 mg/m^3^ → 1.83 mg/m^3^; Liu et al.^28^: 0.5, 3 mg/m^3^ → 0.96, 5.77 mg/m^3^; Li et al.^27^: 0.5, 3 mg/m^3^ → 1.14, 6.86 mg/m^3^; Jung et al.^32^ and Kim et al.^50^; 5, 10 ppm → 5.64, 11.38 mg/m^3^). Thus, based on these studies and their results, we exposed minipigs to 2.16, 4.62, and 10.48 mg/m^3^ at 2 h/day for 2 weeks (5 days a week).

To investigate the effect of FA exposure on physiological parameters, the body weights and relative organ weights (including the lung, spleen, and thymus) of the Yucatan minipigs exposed to FA were assessed. Our data showed that exposure to FA caused no difference in body weight, weight gain, and the relative weight of various organs. These findings are consistent with those of previous studies^30,32^. Additionally, to examine the effect of FA exposure on airway inflammation, the total and differential cell counts of macrophages, eosinophils, neutrophils, and lymphocytes in the bronchoalveolar lavage fluid were determined and histopathological analyses were conducted (data not shown). No significant differences were observed between the total and differential cell counts of the FA-exposed and control groups. These findings are consistent with those of previous studies showing that exposure to FA does not induce significant differences in the counts of various inflammatory cell types in bronchoalveolar lavage fluid^30,46^. However, in the histopathological analysis, infiltration of inflammatory cells and degeneration of the bronchial epithelium were noted to be increased in the 10.48 mg/m^3^ FA exposure group. These observations suggested that the FA concentrations used in this study caused minimal airway inflammation but did not induce direct lung injury. Thus, in the present study, we investigated changes in the splenic immune response at FA concentrations that did not directly promote lung injury.

The spleen is a highly organised lymphoid organ and is important for innate and adaptive immune responses^55^. In the spleen, the proper differentiation and development of different subsets of effector T cells (Th1, Th2) and Treg cells are initiated in the presence of lineage-specific effector cytokines during T cell activation^56^. Recent human and animal studies have reported that FA exposure adversely affects the immune system by altering the population of different types of T cells as well as the production of helper T cell-related cytokines^29,33–35,46^. Thus, we investigated the potential effect of FA exposure on splenic immune responses by evaluating the expression of helper T cell-related mRNAs and cytokines in splenocytes that were activated by Concanavalin A, which induces the mitogenic activity of T cells and increases the synthesis of cellular products. Our results demonstrate that FA exposure suppressed the expression of all helper T cell-related genes in a concentration-dependent manner, while *IL-4* expression was significantly decreased at all inhaled concentrations. These findings are consistent with the results of our previous study, which showed the suppression of Th-1, Th-2, and Th-17 cell-related splenic cytokine production and mRNA expression due to FA exposure in a concentration-dependent manner^46^. Furthermore, Wei et al. reported that levels of helper T cell-related cytokines were suppressed in FA-exposed C57BL/6 mice^29^. Recent studies have revealed that FA exposure suppressed Th1- and Th2-related cytokines in rodent models with ovalbumin sensitisation, thereby resulting in a decrease in airway inflammation and bronchial hyper-responsiveness^27,30^. These results indicate that FA exposure suppresses effector T cell activity, inducing decreased T cell-related mRNA expression and cytokine production.

Treg cells actively suppress pathological and physiological immune responses, which contribute to the maintenance of immunological self-tolerance and immune homeostasis^57^. The suppressive functions of Treg cells can be grouped into four modes of action: (1) suppression mediated by the cytokines IL-10, IL-35, and TGF-β; (2) suppression by cytolysis mediated by granzyme A or B; (3) suppression by metabolic disruption mediated by high-affinity CD25 and cyclic AMP; and (4) suppression by targeting dendritic cells through LAG3 and CTLA4^58^. Hence, to determine whether FA exposure suppresses immune responses via Treg cells, we evaluated the population of helper T cells and Treg cells and evaluated the changes in their signalling pathways. Our results show that exposure to FA caused no difference in the population percentage of CD4^+^ helper T cells in minipigs. However, exposure to FA significantly increased the population size of splenic CD4^+^ Foxp3^+^ Treg cells. Thus, our findings are consistent with those of prior studies on FA-exposed human and rodent models^32,46,59,60^.

Recent studies have indicated that the NFAT-mediated TCR signalling pathway contributes to the induction of Foxp3 expression, which controls the differentiation and function of Treg cells^42^. NFAT proteins are activated by cell surface receptors that are coupled to Ca^2+^ mobilisation^61^. The increased levels of cytosolic calcium are bound by calmodulin, which in turn activates calcineurin, a calcium- and calmodulin-dependent serine/threonine protein phosphatase^62^. NFAT proteins are dephosphorylated by activated calcineurin, resulting in nuclear translocation of these proteins and the induction of NFAT-mediated gene transcription^63^. Recent in vivo and in vitro studies have revealed that NFAT1 plays a crucial role in the suppressive function of Treg cells^64–67^, along with enhancing and maintaining stable Foxp3 expression^62,68–71^. In contrast, NFAT2 induces the activation of effector T cells and the production of effector cytokines in the immune system^72–74^. Our results show that exposure to FA resulted in an increase in NFAT1 expression and Treg cell population size in minipigs, coupled with a decline in IL-4 production. In contrast, FA exposure precipitated no notable difference in NFAT2 expression. These results indicate that FA exposure activated the NFAT-mediated TCR signalling pathway with divergent expression of NFAT1 and NFAT2, leading to an increase in the population of Treg cells. These events may have subsequently induced an immunosuppressive microenvironment along with inhibition of effector T cell activity.

Owing to their close sequence homology with humans, minipigs are considered a useful non-rodent animal model platform for conducting toxicology research. In this study, we evaluated the effects of FA exposure on splenic immune responses in Yucatan minipigs. Our results revealed that exposure to FA increased the differentiation of Treg cells via the NFAT-mediated TCR signalling pathway with divergent expression of NFAT1 and NFAT2, resulting in the suppression of effector T cell activity with decreased production of T cell-related cytokines. Although some studies have reported that FA exposure may provoke or exacerbate Th2-type responses in murine and human models, other studies have found that FA exposure does not aggravate allergic responsiveness and that FA exposure reduces the development of allergic lung inflammation. The differences in species and strains of animals, concentrations and durations of FA exposure, and experimental protocols result in disparate immune responses being observed in response to FA exposure. Therefore, further studies under various conditions (28 days or 90 days for long-term studies; with administration of low and high doses of FA) are necessary to determine the impact of FA exposure on the immune systems. In conclusion, our findings provide insight into the molecular mechanisms underlying the FA exposure-induced development of an immunosuppressive microenvironment, characterised by an increased Foxp3^+^ Treg cell population. Development of such an immunosuppressive microenvironment may potentially result in detrimental health effects, such as increasing host susceptibility to opportunistic infections and the progression of cancer.

## Methods

### Animals

Six-month-old male Yucatan minipigs (Optipharm Inc., Chenongju, Korea) were used in this study. Each minipig was placed in an individual pen in an animal room with controlled temperature (22 ± 2 °C) and humidity (50 ± 5%), a 12 h light/dark cycle, and positive pressure with HEPA-filtered air. The animals were fed sterilised food pellets (Farm Story Dodram B&F, Seoul, Korea) and sterilised tap water ad libitum and were acclimatised for 3 weeks prior to commencement of FA exposure. All experimental procedures carried out in this study were reviewed and approved by the Institutional Animal Care and Use Committee of the Korea Institute of Toxicology (IACUC #1908-0297). All procedures were performed in accordance with the relevant guidelines and regulations, including compliance with ARRIVE guidelines.

### Experimental groups

**Figure 5:**
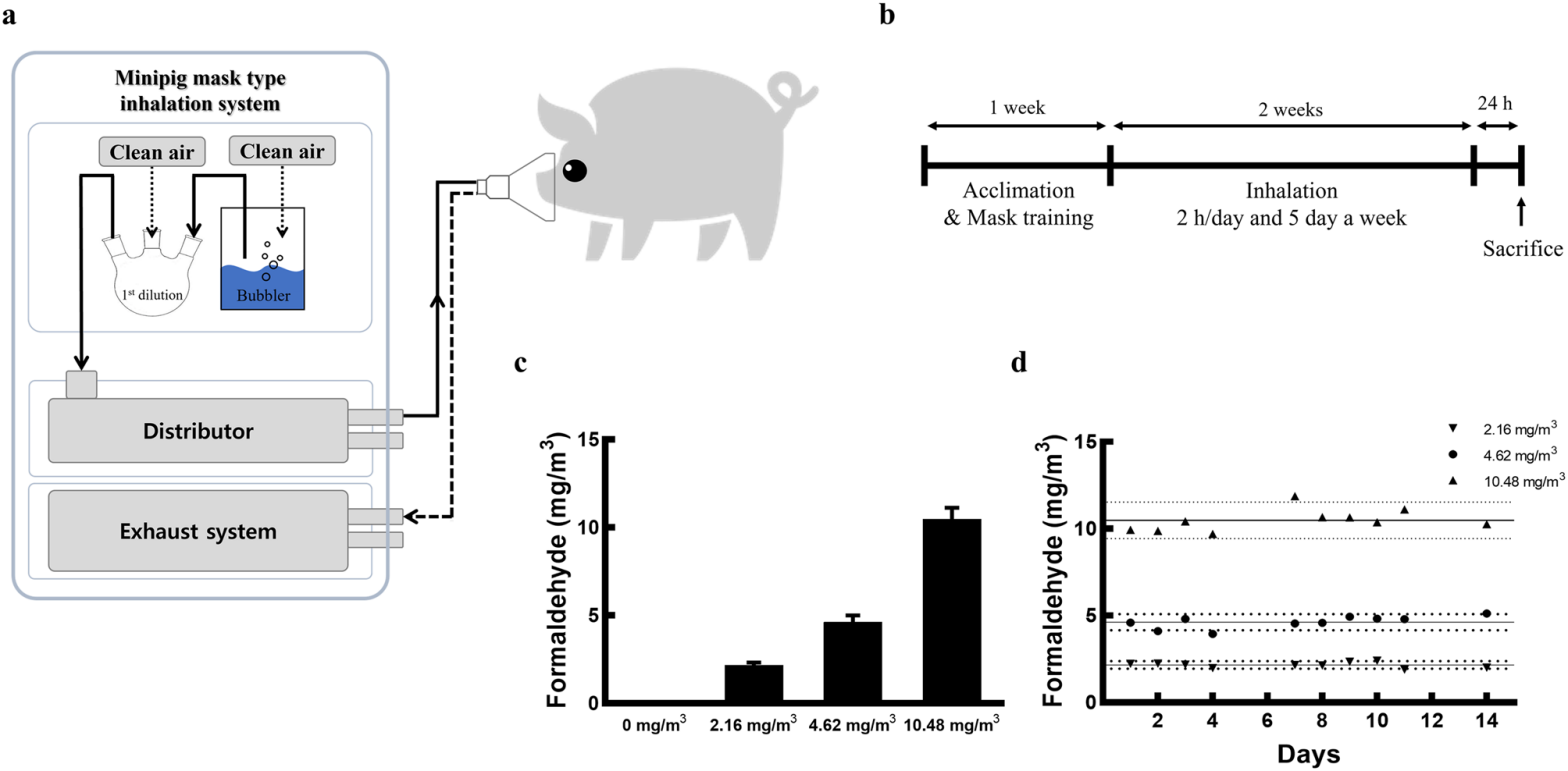
Schematic of the minipig mask-type inhalation system for FA exposure, experimental design, and FA concentrations for assessing the effect of FA exposure on minipigs. (**a**) FA was generated using a gas bubbler and diluted with clean air using a multi-neck flask. (**b**) All minipigs received mask training for 2 h per day before FA exposure. Minipigs in the treatment groups were exposed to FA for a period of 2 weeks at 2 h/day, 5 days a week using the minipig mask-type inhalation system. The (**c**) mean and (**d**) daily concentrations of FA exposure were monitored using a Top Solid DNPH cartridge and HPLC system. The groups of Yucatan minipigs were exposed to 0 mg/m^3^ (control), 2.16 ± 0.16 mg/m^3^, 4.62 ± 0.36 mg/m^3^, or 10.48 ± 0.64 mg/m^3^ FA for 2 weeks (5 days/week) at 2 h/day. Data are presented as the mean ± SD. *FA* formaldehyde.

The minipigs were randomly divided into four groups (*n* = 2 minipigs/group): control group, 2.16 mg/m^3^ FA exposure group, 4.62 mg/m^3^ FA exposure group, and 10.48 mg/m^3^ FA exposure group. The control minipigs were not exposed to FA. Minipigs in the treatment groups were exposed to FA for a period of 2 weeks at 2 h/day for 5 days a week through a minipig mask-type inhalation system (Fig. 5a,b). Body weights were measured on days 2, 6, 9, and 13 prior to FA exposure. Terminal body weight was measured 24 h after the last FA exposure. The pigs were sedated using 0.5 mg/kg midazolam and 5 mg/kg ketamine intramuscularly and were euthanised under isoflurane anaesthesia. All samples were collected for subsequent analysis.

### FA exposure

FA was administered as a methanol-free ultrapure 10% FA solution (Polysciences Inc., Warrington, PA, USA) using the minipig mask-type inhalation system. The FA was diluted with clean air using a multi-neck flask to achieve the desired FA concentrations and delivered through the minipig mask (Fig. 5a). The FA in the mask was sampled using a Top Solid DNPH cartridge (Top-Trading Co., Seoul, Korea) and was monitored hourly by high-performance liquid chromatography with ultraviolet detection (HPLC–UV). All of the minipig groups were exposed to 0 (control), 2.16 ± 0.16 (mean ± SD), 4.62 ± 0.36, or 10.48 ± 0.64 mg/m^3^ FA (Fig. 5c,d).

### HPLC–UV analysis

The FA-2,4-dinitrophenylhydrazine (FA-2,4-DNPH) derivative was analysed on an Agilent 1260 Infinity HPLC system (Agilent Technologies, Santa Clara, CA, USA) equipped with a degasser (Agilent 1260 Infinity series, G1322A), a binary pump (Agilent 1260 Infinity series, G1312B), an autosampler (Agilent 1260 Infinity series, G1329B), a thermostat column compartment (Agilent 1260 Infinity series, G1316B), and a diode array detector VL (Agilent 1260 Infinity series, G1315D). A Gemini 5 µm C18 110A column (length, 150 mm; diameter, 4.6 mm; particle size, 5 μm; Phenomenex, Torrance, CA, USA) was used as the analytical column. Acetonitrile/ distilled water (60:40, v/v) was used as the mobile phase. The injection volume was 10 μL, flow rate was 1.0 mL/min, and column temperature was 40 °C. The analyte was monitored at a wavelength of 360 nm. Quantitation was performed using a synthesised FA-2,4-DNPH solution (Sigma-Aldrich Co., St. Louis, MO, USA) as the standard.

### Spleen cell preparation and culture

The spleen was isolated from each minipig exposed to FA. Single splenic cells were prepared as described previously^46^. For primary cell culture, the single splenic cells were suspended in RPMI 1640 medium (Lonza, Walkersville, MD, USA) containing 10% heat-inactivated foetal bovine serum (Gibco, Grand Island, NY, USA), penicillin (100 U/mL) (Gibco), and streptomycin (100 μg/mL) (Gibco). The splenocytes were seeded at a density of 1 × 10^6^ cells/100 µL/well in 12-well culture plates (Eppendorf, Hamburg, AG, Germany) and incubated for 72 h with 2.5 μg/mL Con A (Sigma-Aldrich Co.) at 37 °C with 5% CO_2_.

For flow cytometric analysis, the splenic single cells were resuspended in Flow Cytometry Staining Buffer (eBioscience Inc., San Diego, CA, USA) prior to analysis.

### Quantitative reverse transcription-polymerase chain reaction (qRT-PCR)

Total RNA was extracted from cultured splenocytes using the RNeasy Mini Kit (Qiagen, Valencia, CA, USA) according to the manufacturer's protocol and quantified using the QIAxpert system (Qiagen). Complementary DNA was synthesised by reverse transcribing total RNA (400 ng) using the GoScript™ Reverse Transcription system (Promega, Madison, WI, USA) in a MasterCycler^®^ Nexus GX2 thermal cycler (Eppendorf). The primer sequences were as follows: minipig *GAPDH*, 5′-GGGCATGAACCATGAGAAGT-3′ and 5′-TGTGGTCATGAGTCCTTCCA-3′; *IL-4*, 5′-AGAACACGACGGAGAAGGAA-3′ and 5′-TTGCCATGCTGCTAGGTT-3′; *IFN-γ*, 5′-CAGCTTTGCGTGACTTTGTG-3′ and 5′-TTTTGTCACTCTCCTTCCAAT-3′; and *TNF-α*, 5′-CCCCCAGAAGGAAGAGTTTC-3′ and 5′-CGGGCTTATCTGAGGTTTGA-3′. The qRT-PCR was performed on a QuantStudio™ 5 Real-Time system (Thermo Scientific, Wilmington, DE, USA) with Power SYBR Green Master Mix (Thermo Scientific). The transcript level for each gene was normalised to that of *GAPDH*.

### Cytokine production of spleen cell cultures

The splenocyte culture medium, obtained 72 h after primary splenocyte culture, was assessed for the cytokines IL-4, IFN-γ, and TNF-α using the ProcartaPlex™ immunoassay kit (eBioscience) and the Luminex 200™ system (Luminex Corporation, Austin, TX, USA) according to the manufacturer's instructions. Cytokine concentrations were quantified by evaluating the fluorescent signal of analyte-specific capture beads and analysed using ProcartaPlex Analyst 1.0 (eBioscience). All standards and samples were measured in duplicate.

### Flow cytometric analysis

The single spleen cells isolated from FA-exposed minipigs were washed with Flow Cytometry Staining Buffer (eBioscience) and stained with PerCP-Cy™5.5-conjugated anti-CD4 monoclonal antibody (BD Biosciences, San Jose, CA, USA) for 60 min on ice. For intracellular staining, the spleen cells were permeabilised with a Fixation/Permeabilization solution (eBioscience) and stained with an FITC-conjugated anti-Foxp3 monoclonal antibody (eBioscience) for 60 min at 20 °C. All samples were run on a CytoFlex flow cytometer (Thermo Scientific). Data of 50,000 events were collected and analysed using CytoExpert 2.3 software (Thermo Scientific).

### Western blot analysis

The spleen tissue was processed in RIPA buffer (Pierce Biotechnology, Rockford, IL, USA) to generate denatured protein lysate, which was quantified using the Pierce BCA Protein Assay kit (Thermo Scientific). Protein lysates (30 µg) were mixed with 4 × Laemmli sample buffer (Bio-Rad Laboratories, Hercules, CA, USA), separated by 8% SDS-PAGE, and transferred onto polyvinylidene difluoride (PVDF) membranes (Millipore, Billerica, MA, USA). The membranes were blocked using 5% bovine serum albumin in Tris-buffered saline with 0.1% Tween 20 (TBS-T) for 60 min at 20–24 °C The membranes were then incubated with anti-NFAT1, anti-NFAT2 (Abcam, Cambridge, UK), and anti-β-actin antibodies (Santa Cruz Biotechnology, Santa Cruz, CA, USA) overnight at 4 °C. After washing thrice with TBS-T, the membranes were incubated for 60 min at 20–24 °C with horseradish peroxidase-conjugated goat anti-rabbit and anti-mouse IgG (Santa Cruz Biotechnology). After washing thrice with TBS-T, the immunoreactive signals were visualised with chemiluminescent reagents (Pierce Biotechnology) using the ChemiDoc MP Imaging system (BD Biosciences). Colorimetric detection also performed. The densitometric analysis results for specific protein signals were quantified using Image J 1.51 K (National Institutes of Health, Bethesda, MD, USA). All results were normalised to the expression of β-actin. The original raw data are shown in Supplementary information.

### Statistics

All data are presented as the mean ± SD from two independent experiments. Statistical analysis was performed using the Statistical Package for the Social Sciences (Version 25; SPSS, Chicago, IL, USA). Differences between groups were evaluated by one-way ANOVA and the Kruskal–Wallis test. Tukey's tests and Bonferroni-adjusted Mann–Whitney *U* tests were used as the post-hoc tests. *p* < 0.05 was considered statistically significant.

## Supplementary Information

Supplementary Information.

## Data Availability

All data generated or analysed during the current study are accessible from the corresponding author upon reasonable request.
