# Supplementary figures and images for: Formaldehyde exposure induces differentiation of regulatory T cells via the NFAT-mediated T cell receptor signalling pathway in Yucatan minipigs
Source: Sci Rep. 2022 May 17;12:8149. doi: 10.1038/s41598-022-12183-8 (PMC9114421; doi:10.1038/s41598-022-12183-8)

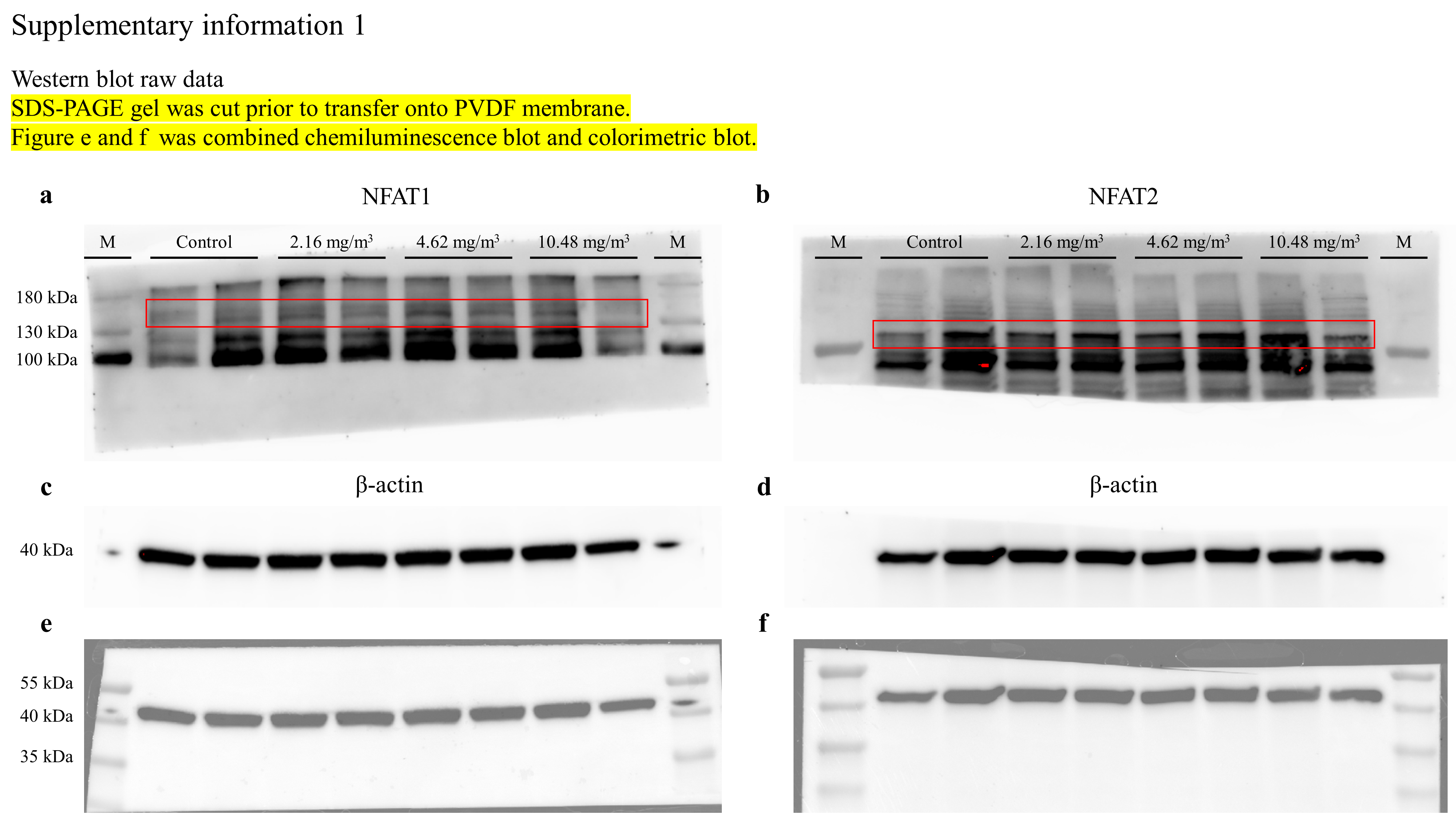

Supplement: Supplementary file 1 — Supplementary Information. [file 41598_2022_12183_MOESM1_ESM.tif]
